# Conservation Status of a Recently Described Endemic Land Snail, *Candidula coudensis*, from the Iberian Peninsula

**DOI:** 10.1371/journal.pone.0138464

**Published:** 2015-09-17

**Authors:** Francisco Moreira, Gonçalo Calado, Susana Dias

**Affiliations:** 1 Department of Life Sciences, Lusófona University, Campo Grande, Lisbon, Portugal; 2 REN Biodiversity Chair, CIBIO/InBIO Associate Laboratory, Uxniversidade do Porto, Campus Agrário de Vairão, Vairão, Portugal; 3 Centro de Ecologia Aplicada Prof. Baeta Neves/InBIO Associate Laboratory, Instituto Superior de Agronomia, Universidade de Lisboa, Tapada da Ajuda, Lisbon, Portugal; Australian Museum, AUSTRALIA

## Abstract

We assessed the distribution, population size and conservation status of *Candidula coudensis*, a recently described endemic land snail from Portugal. From March 2013 to April 2014, surveys were carried out in the region where the species was described. We found an extent of occurrence larger than originally described, but still quite small (13.5 km^2^). The species was found mainly in olive groves, although it occurred in a variety of other habitats with limestone soils, including grasslands, scrublands and stone walls. Minimum population estimate ranged from 110,000–311,000 individuals. The main identified potential threats to the species include wildfires, pesticides and quarrying. Following the application of IUCN criteria, we advise a conservation status of either “Least Concern” or “Near-threatened” under criterion D (restricted population).

## Introduction

Land snails of the Iberian Peninsula are quite diverse, with some clades reaching high levels of endemism [1, 2]. The genus *Candidula* Kobelt, 1871 (Hygromiidae) is one such example, with 10 out of 13 species being endemic [3] and new species still being found [4]. *Candidula coudensis* G. Holyoak & D. Holyoak, 2010 is one of the most recently described species in the genus. Endemic to Portugal, it is a highly distinctive species with a discoidal dextral shell, 5–11 mm diameter, strongly compressed and with a sharp keel around the body whorl [4, 5]. The only similar species that could lead to misidentification of *C*. *coudensis* is another Portuguese endemic, *Candidula setubalensis* L. Pfeiffer, 1850. However the 2 species are allopatric, separated by a gap of 150 km, with *C*. *setubalensis* occurring in a very narrow and well defined geographic area, and having distinctive features (a much shorter penial flagellum and paler coloration of external parts of the body) [4]. Holyoak & Holyoak (2010) reported *C*. *coudensis* from an area of less than 1 km^2^ in central Portugal [5] along a limestone ridge with dwarf scrublands, olive groves and other crop fields surrounded by limestone walls, and patches of Lusitanian Oaks (*Quercus faginea*). Considering the scarce information available from the original paper, we undertook a survey with three aims: (1) assessing the distribution of the species and making a preliminary estimation of population size; (2) identifying potential threats to the species; (3) applying IUCN criteria to advise a conservation status category. This latter aim could be used by IUCN assessors as a guideline to propose an official conservation status to *C*. *coudensis*.

## Methods

### Study area

A study area of around 10,800 ha was located in central Portugal, Leiria district (39°50’N, 8°25’ W), and centered on the type-locality (Vale da Couda) [5]. Altitude varies from 100 m to 618 m with the highest hilltops at the summit of Alvaiázere ridge. The climate is Mediterranean, moderately humid, mesothermic with mean annual rainfall of 1000 mm [6] mostly concentrated in spring and autumn. The substrate is dominated by limestone, thus the landscape is mostly rugged with narrow valleys, rocky outcrops and bare stony slopes. Xerophilous habitats with sparse vegetation dominate the hill tops. Slopes are occupied by dwarf shrubs with patches of dense scrub dominated by *Pistacia* and *Cistus*. Lowlands are used mainly for annual crops and olive orchards, but in the hills, traditional olive groves have been gradually invaded by Mediterranean shrubs (due to land abandonment) or replaced by pines (*Pinus pinaster*) and exotic eucalyptus trees (*Eucalyptus globulus*). Patches of Lusitanian Oak (*Quercus faginea)* forest still persists. Extensive grazing (mainly by goats) still occurs in the area. Limestone walls are a common feature in both the rural areas and small villages. The area is included in a Natura 2000 site (Sicó Alvaiázere—PTCON0045) due to its importance for limestone habitats (as an example of karst geomorphology) and plant species, harboring relict *Quercus* forests along with rare and endangered orchids and other bulbous plants (e.g., *Narcissus calcicola*). This area is prone to fast-spreading fires which occur during hot, dry summers. In the period 1978–2014, a total of 9100 ha were burned by 90 wildfires, with the single largest event burning 1000 ha in 2005 (data from the Portuguese Fire Atlas [7]).

### Ethics Statement

We only collected dead shells in the field. No living specimens were collected or sacrificed. Samples were taken on communal land and private properties. No specific permission was required for crossing these areas or carrying out snail surveys. However, when land owners were known, they were contacted and permission was granted. The target species is not protected by any national law or local regulation.

### Geographic distribution patterns and habitat use

**Fig 1:**
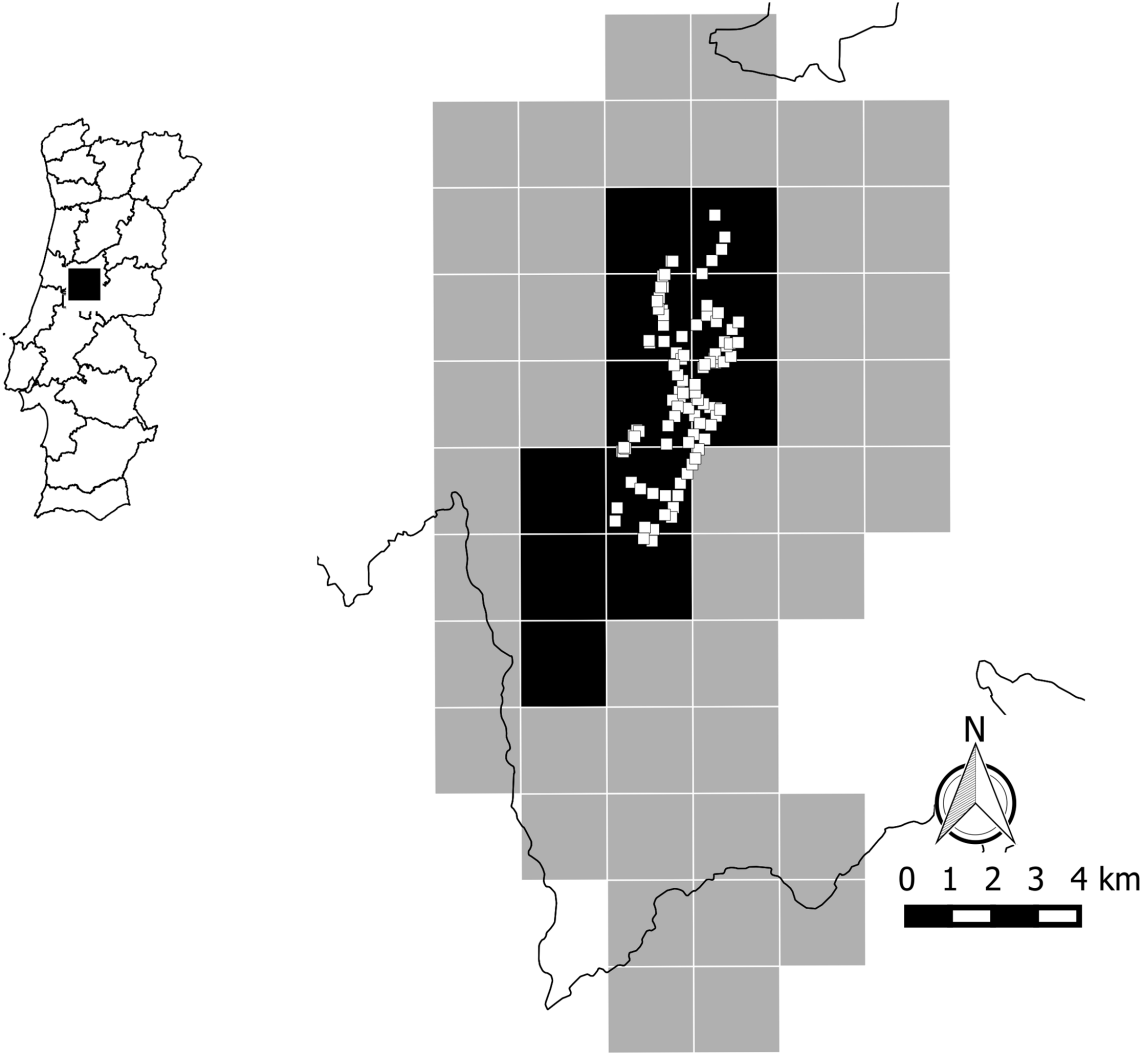
Study Area. Map of Portugal and study region (left), and detailed information (right) on 2 x 2 km tetrads used to sample the land snail *Candidula coudensis*. Black grid squares indicate presence, grey squares indicate absence of either live individuals or shells. Within black squares, small white squares correspond to locations of 5 x 5 m quadrats used to assess population density. The type-locality where the species was described is located at near the center of the grid.

To identify the extent of occurrence of the species, starting from the type locality described in [5], we set a grid of fifty four 2 km^2^ tetrads (grid squares centered on the type locality) in the surrounding region, totaling 108 km^2^ (Fig 1). Each tetrad was searched for 15–40 minutes, at 1 to 3 different locations (preferably in apparently suitable habitat, if available), by a group of 10–15 volunteers (Lusófona University students and teachers from the Biology course), during March to December 2013, searching suitable micro-habitats in each location (on and under stones, inside crevices, in stone walls, in herbaceous and scrub vegetation). The presence or absence of live specimens or dead shells in each of the tetrads was recorded with GPS coordinates and subsequently incorporated into a Geographic Information System (GIS). The land cover types where the species was recorded were determined *a posteriori* by overlaying occurrence locations with the CORINE land cover map [8] in the GIS. The same was done for altitude, rainfall, and soil type data available from the Portuguese Atlas of Environment [6].

### Population estimates

Based on the previous task, 97 quadrats of 25 m^2^ each (5 x 5 m) were spread within the 2 km^2^ tetrads where live animals were known to occur (Fig 1). These were systematically spread across the whole species range, along available roads and tracks. Minimum distance between quadrats ranged from 32 to 555 m (mean = 177 m). These 97 sites were visited once between December 2013 and April 2014 (corresponding to the rainy season, when snail activity is likely to be greatest [9, 10]) by groups of 4–5 people, with all live animals and empty shells within the quadrat being recorded. Density estimates were derived by direct extrapolation (dividing the total number of individual snails by the quadrat area), thus they were not adjusted for detectability. Snail density was assumed to be homogeneous within its distribution area, so that population size (with an associated error according to S.E. of the mean) could be estimated by multiplying the overall mean density by total distribution area. Because of these methodological limitations (no detectability corrections, “snapshot”census, and snail densities assumed to be homogeneous), population densities and the population size derived from them should be considered with caution, and are likely to represent minimum estimates.

### Assessing the conservation status and threats

The conservation status of *C*. *coudensis* was assessed by applying the IUCN criteria, created to provide an explicit, objective framework for the classification of the broadest range of species according to their extinction risk [11, 12]. IUCN classification is based on a set of five criteria (A—Population reduction, B—Geographic range, C—Small population size and decline, D—Very small or restricted population, E—quantitative analysis of extinction risk. The species was evaluated using all the IUCN criteria and the criteria for the highest category that the species qualifies listed [12]. Extent of occurrence (EOO) was measured by a minimum convex polygon (the smallest polygon in which no internal angle exceeds 180 degrees and which contains all the sites of occurrence) [11]. This was estimated separately for the whole set of data (locations of live animals and dead shells) and using only the locations of live specimens. Area of occupancy (AOO) is the area within the extent of occurrence which is occupied by the species [11]. For assessing likely threats to the species, we used the IUCN Threats Classification Scheme (version 3.2).

## Results and Discussion

### Geographic distribution pattern

**Fig 2:**
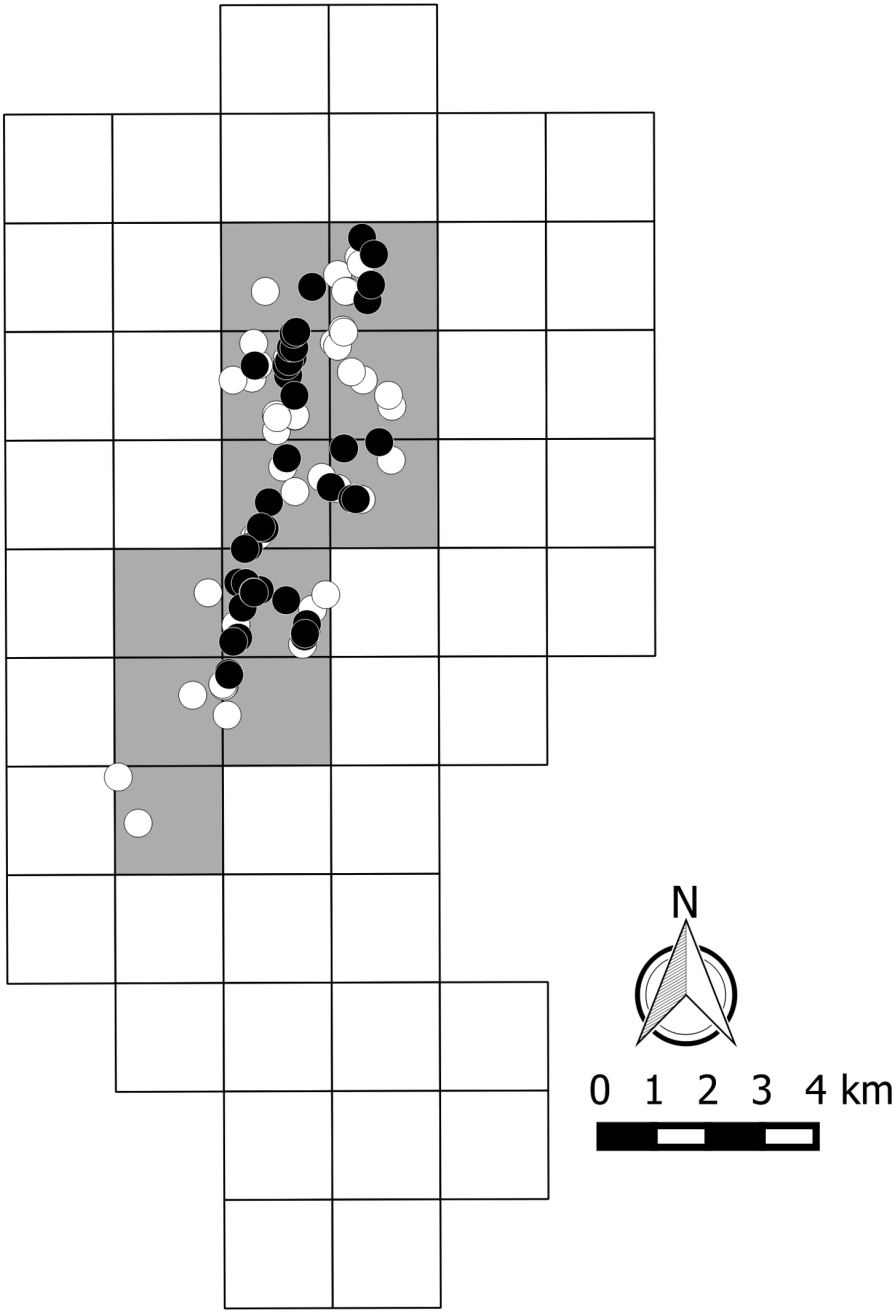
Occurrences of *Candidula coudensis*. Location of living *Candidula coudensis* (black dots) and empty shells (white dots) within the 2 x 2 km grid used to sample this land-snail species. Grey grid squares correspond to species presence (either live or dead).

Of the fifty four 2 x 2 km tetrads prospected in 2013, the species was found in 11 (Fig 1). Within this area, a total of 89 different locations was recorded (Fig 2). The extent of occurrence (EOO) of the species (live specimens or empty shell remains) was 25.2 km^2^. However, the EOO of live animals was smaller (13.5 km^2^) and fully nested within the former area. The Area of Occupancy (AOO) was considered equivalent to the EOO, as live individuals seemed to be widely distributed within the latter, and the apparent absence in a few areas (e.g. hilltops, pine plantations) might have been due to low densities/detectability.

### Habitat use and environmental correlates

Using CORINE land cover categories, the species was mostly found on “olive groves” (33 locations), followed by “Land principally occupied by agriculture, with significant areas of natural vegetation “(20), “Sclerophyllous vegetation” (16), “Transitional woodland-shrub” (10), “Natural grasslands” (8) and “Discontinuous urban fabric” (2). This shows a broad range of habitats used permanent crops, forest patches, grasslands and scrub, and even stone walls within villages. It was commonly found on or under stones, and we have also found it in stone walls, on the soil surface and in herbaceous (more rarely in scrubby) vegetation. The species seemed most common in herb-rich grasslands in traditional olive groves. Data from the Portuguese Atlas of Environment [6] showed that annual rainfall in the EOO is 1200–1400 mm per year. Altitude at registered locations ranged from 148 to 524 m (mean = 334.5 m). The vast majority of records (95.5%) were in calcium carbonate rich soils or among exposed limestone rocks [6].

### Population estimates

Of the 97 sampled 25 m^2^ quadrats, *C*. *coudensis* was recorded in 26 (26.8%), of which 15 had both live animals and empty shells, 7 only had empty shells and 4 only had live animals. Where they occurred, the number of live animals usually ranged from 1 to 5. In one quadrat, however, we found 26 live snails. This unusually rich sample was treated as an outlier and excluded from the subsequent analyses. There was no significant correlation between the number of live and dead animals found across quadrats (Spearman r = 0.24, p = 0.245, n = 25). The average density (± standard error) of live snails in the sampled quadrats was 0.39 ±0.095/25m^2^ (n = 96). Extrapolating this density to an area of 13.5 km^2^ yielded a total population estimate of 110,052–311,148 individuals. This interval should be considered a minimum estimate, as no detectability corrections were made.

### Potential threats to the species

Based on the IUCN threat classification scheme (version 3.2), the following main potential threats to the population were identified during field work:

Wildfires—This is the only risk factor that could potentially affect a significant part of the distribution range in a single event. However, more research is needed to assess the impact of wildfires on this snail. Fires may affect snails directly by incineration, by dehydration and contamination by pollutants released during the combustion of the organic layer, but the importance of each factor is unknown. Fires will also affect the quality of the snail's habitat [13, 14]. Wildfires occur mainly in the dry season, a period when individual snails are often found aestivating deep in crevices and other hidden sites. Their depth may provide enough protection during low-intensity fires which often occur in habitats dominated by boulders and rock outcrops [15]. Furthermore, fire patterns in the distribution area of *C*. *coudensis* usually leave unburned or lightly burned patches that provide refuges [10, 14, 16] in which the species can survive. Hence, as with other Mediterranean land snails, *C*. *coudensis* is expected to show some resilience to fires provided that the time between successive disturbances is longer than the time required for population recovery [9]. Fires probably have a dual role, as they also maintain open habitats in the area by averting scrub encroachment or woodland closure, thus preventing an increased area of habitats apparently less suitable for the species. Therefore, we assume that wildfire impacts could potentially range from moderate to strong, presumably increasing now because of the likelihood of expansion of the fire season and severity due to climate change [17, 18].

Herbicides and other pesticides—Direct observations confirmed that agro-chemicals are often applied in at least some olive groves, mainly to control herbaceous vegetation and facilitate olive picking (which often relies on sheets or nets placed on the ground). Depending on the spatial extent of this practice, it will limit likely food resources and habitat quality of *C*. *coudensis*, leading to potentially moderate to strong impacts (at least at local level).

Quarrying–*C*. *coudensis* occurs in a region where limestone is quarried to provide stone and gravel. Within its distribution area small quarries are in operation and these doubtless affect habitats such as rocky outcrops and deep crevices. Besides direct habitat destruction and death of some specimens, dust from the excavation can adversely affect the animals [1]. This impact is potentially moderate.

Renewable energy—Most hill tops in the region have wind farms, which required the installation of collector systems and substations and access roads to each turbine site. This presumably caused habitat loss. This impact is currently considered potentially weak to moderate.

Housing and urban areas—trends of continuous abandonment of small villages are evident in the region, thus habitat destruction due to urbanization is not foreseen in the area. Although construction can adversely affect some populations in the short term, the species as a whole can survive in unaffected regions. Furthermore stone walls, still abundant in the area, are microhabitats used by the species, so the real threat may be from replacement of traditional stone walls by concrete block walls or fences. This impact is currently considered potentially weak.

Roads—Road construction and use may have moderate impacts depending on their extent and traffic intensity. During construction, especially in steep-sided valleys, beside direct destruction of snails, blasting changes rock faces, leaving them potentially unsuitable for the snails for a long time. However, *C*. *coudensis* was frequently found in open disturbed ground near road sides. Therefore the species may colonize rocks in newly opened areas resulting from blasting. Due to its low dispersal ability (like most other land-snails [19]), roads may contribute to habitat fragmentation and reduce connectivity between sub-populations [20]. No significant development of new roads in the area is expected in the future, thus this impact is considered potentially weak to moderate.

Climate change—Droughts are expected to become increasingly frequent in the Mediterranean region [17, 21]. Land snails are generally very sensitive to desiccation, although xerophilous species like *C*. *coudensis* are adapted to withstand drought e.g. by confining most activity to periods of high air humidity, particularly during the winter, which correspond in general with the reproductive season [5]. Longer droughts may lead to less activity and longer aestivation periods with possible adverse effects on finding mating partners (and hence on egg laying success, [22]) and feeding. This impact is considered potentially weak to moderate.

### Assessing the conservation status

Criterion A (population reduction) could not be applied, as this was the first time the species was extensively surveyed. Consequently, we do not have data on population and range size changes. In addition, the species does not seem to be a habitat specialist; therefore, no changes could be inferred from modifications in the extent of particular habitat types. For criterion B (geographic range), three conditions have to be met simultaneously: small range together with habitat fragmentation or reduced number of locations, continuing decline or extreme fluctuations. Although the EOO was less than 100 km^2^, there were no data providing evidence of a continuing decline or the existence of extreme fluctuations in population. In spite of a presumably low dispersal capability [19, 23] the distribution appeared to be roughly continuous within the overall range. If there is fragmentation of the occupied habitats resulting in numerous isolated sub-populations, populations within the different habitat patches appear to be viable at present. As for the number of locations, based on the IUCN definition of location as ‘‘a geographically or ecologically distinct area in which a single threatening event can rapidly affect all individuals of the taxon present”, we consider that tens of locations probably occur in the region, assuming that wildfires will not drastically affect the whole population (see above). Hence, the species did not acquire any threat status under this criterion. For criterion C (small population size and decline) our population estimate, likely to be an underestimate, is much larger (even if the proportion of adult individuals is unknown) than 10,000 individuals, so the species should not be classified as threatened. However, Cardoso et al. [24] claim that IUCN thresholds of abundance for invertebrates should be increased, probably by a few orders of magnitude. For criterion D (very small or restricted population), although the area of occupancy was less than 20 km^2^, it is not plausible that the species could become Critically Endangered or Extinct in a very short time period, as a consequence of human activities or stochastic events, therefore the species could be classified as “Least Concern”. However, IUCN guidelines state that “If the taxon is highly restricted, and there are plausible threats that can cause the species to become VU or EN in a short time, then the taxon should be considered for listing as NT” [12] (page 59]. Therefore, if it is considered plausible that a large wildfire could strongly affect a significant part of the population, causing a (suspected) population decline of over 50% (Criteria A1), then the species could also be considered for listing as “Near-Threatened”. Criterion E (Quantitative analysis of extinction risk) was inapplicable because the assessment requires unavailable data for carrying out a Population Viability Analysis or other quantitative analysis.

## Conclusions

Compared to the range originally reported of less than 1 km^2^ [5], we found that *C*. *coudensis* occurred in a much larger area, although it was still small (less than 20 km^2^). While the eastern and western limits of distribution of the species seem to be dictated mainly by the limits of the limestone strip that extends in a north-south direction, there seem to be no obviously distinctive geologic or habitat features that could explain the northern and southern edges of its distribution. Altitudinal range was also found to be larger than initially reported (150–500 m, compared to 380–390 m [5], but extended to 263–456 m with additional records [4](p. 667). The association of the species with managed habitats (olive groves, grasslands, low scrub) suggests it can cope with or even benefit from disturbance events, as described for other snails (e.g. [25, 15]). Further research is nevertheless needed on its ecological requirements, and in particular, on the impact of wildfires, which could constitute the only threat potentially affecting most of its range. Likewise, the impacts of herbicide use in olive groves should be the focus of research, along with studies on population dynamics, genetics and dispersal capabilities, in order to evaluate potential fragmentation among existing sub-populations. Based on the evidence available, the total population size seems to be in the range of hundreds of thousands of individuals. Following application of IUCN criteria, we propose that the species should be classified as “Least Concern” or “Near-threatened”. The fact that its entire range is located within a Natura 2000 site may facilitate the implementation of management measures and restrictions compatible with the species conservation.

## Supporting Information

S1 Table Data on *Candidula coudensis* abundances. Number of empty shells (dead animals) and live individuals found in each sampled quadrat of 25m^2^.
(XLSX)
